# The Angiogenic Secretome in VEGF overexpressing Breast Cancer Xenografts

**DOI:** 10.1038/srep39460

**Published:** 2016-12-20

**Authors:** Louis Dore-Savard, Esak Lee, Samata Kakkad, Aleksander S. Popel, Zaver M. Bhujwalla

**Affiliations:** 1 JHU ICMIC Program, Division of Cancer Imaging Research, The Russell H. Morgan Department of Radiology and Radiological Science, The Johns Hopkins University School of Medicine, Baltimore, Maryland, USA; 2 Systems Biology Laboratory, Department of Biomedical Engineering, The Johns Hopkins University School of Medicine, Baltimore, Maryland, USA; 3 Sidney Kimmel Comprehensive Cancer Center, The Johns Hopkins University School of Medicine, Baltimore, Maryland, USA

## Abstract

The plasticity of cancer cells and the fluidity of the tumor microenvironment continue to present major challenges in the comprehensive understanding of cancer that is essential to design effective treatments. The tumor interstitial fluid (TIF) encompasses the secretome and holds the key to several of the phenotypic characteristics of cancer. Difficulties in sampling this fluid have resulted in limited characterization of its components. Here we have sampled TIF from triple negative and estrogen receptor (ER)-positive human breast tumor xenografts with or without VEGF overexpression. Angiogenesis-related factors were characterized in the TIF and plasma, to understand the relationship between the TIF and plasma secretomes. Clear differences were observed between the TIF and plasma angiogenic secretomes in triple negative MDA-MB-231 breast cancer xenografts compared to ER-positive MCF-7 xenografts with or without VEGF overexpression that provide new insights into TIF components and the role of VEGF in modifying the angiogenic secretome.

One of the least examined, and yet critically important factors of the tumor microenvironment (TME) is the tumor interstitial fluid (TIF) that contains the tumor secretome^1^. This fluid surrounds cancer and stromal cells and contains various cytokines, and nutritional and molecular factors that shape the outcome of nearly all aspects of tumor angiogenesis, growth, metastasis, and response to treatment. As mining of targets to treat cancer expands into the TME, the TIF also represents an important source of identifying new targets in cancer treatment.

Some of the earliest work in sampling TIF using a collection chamber was performed by Gullino and colleagues^2^. Despite the limited analytical methods that were available at the time, important insights into the protein content, urea, free amino acids, glucose and lactate in TIF were gained^2^. During the last decade, newer TIF isolation methods, and physiological and molecular characterization have been applied to unravel the role of the TIF in cancer^3^,^4^,^5^. Methodologies such as the incubation of human tumor samples in a buffer and subsequent concentration of the samples^6^ have allowed antibody-based analyses of TIF that identified the presence of several cytokines and other proteins. Microdialysis has also been used to collect and analyze tumor-secreted proteins in xenograft models using quantitative high-performance liquid chromatography^7^. This method provided important information about TIF content including overexpression of proteins such as vascular endothelial growth factor (VEGF)^8^,^9^. Factors secreted by breast cancers and their roles in distal metastasis were also recently investigated^4^,^5^,^10^,^11^.

Interpretation of TIF content and its influence on the pathophysiological properties of the tumor, such as angiogenesis, invasiveness and metastasis is limited. A few studies have extensively screened the protein content in TIF^12^,^13^,^14^, but these have primarily been proof-of-principle studies to demonstrate the feasibility of proteomic analysis of the interstitial fluid.

Angiogenesis, one of the major hallmarks of cancer, is essential for cancers to establish vasculature for growth and hematogenous metastasis^15^. Cytokines and growth factors secreted by cancer cells and stromal cells stimulate endothelial cell proliferation^16^. Among all known angiogenic factors, vascular endothelial growth factor is closely associated with increased aggressiveness and metastasis^17^, and is known to be upregulated under hypoxia^18^. Preclinical and clinical studies have demonstrated that VEGF can be targeted to reduce angiogenesis and VEGF blockage has been shown to promote survival – especially when combined with chemotherapies – and slow tumor growth in ovarian, cervical, colorectal, renal, lung and breast cancer^19^. Additionally, levels of VEGF in plasma may be a biomarker of response to anti-angiogenic therapy^20^. Despite recent setbacks in the use of anti-angiogenic therapy in breast cancer, the importance of VEGF in tumor progression, and its potential targeting for treatment remains undeniable^21^,^22^,^23^,^24^.

To further understand the TIF and plasma secretome in breast cancer, and the role of VEGF in modifying these secretomes, we characterized angiogenesis-related cytokines, small proteins and peptides in TIF from triple negative MDA-MB-231 and ER-positive MCF-7 human breast cancer xenografts with and without VEGF overexpression. Triple negative breast cancer is characterized by increased aggressiveness, high recurrence and mortality, and resistance to treatment^25^ compared to ER-positive breast cancers^26^. TIF was obtained using a collection chamber modified for mouse tumors, based on a technique initially described by Gullino and colleagues^2^. Secretome factors in TIF were compared to those in plasma to understand the role of TIF in modifying the plasma factors that may impact metastatic dissemination and the formation of the pre-metastatic niche, and also potentially provide plasma-derived biomarkers.

## Results

### Angiogenesis-related factors in TIF and plasma of MCF-7 wt and VEGF overexpressing tumors

**Figure 1:**
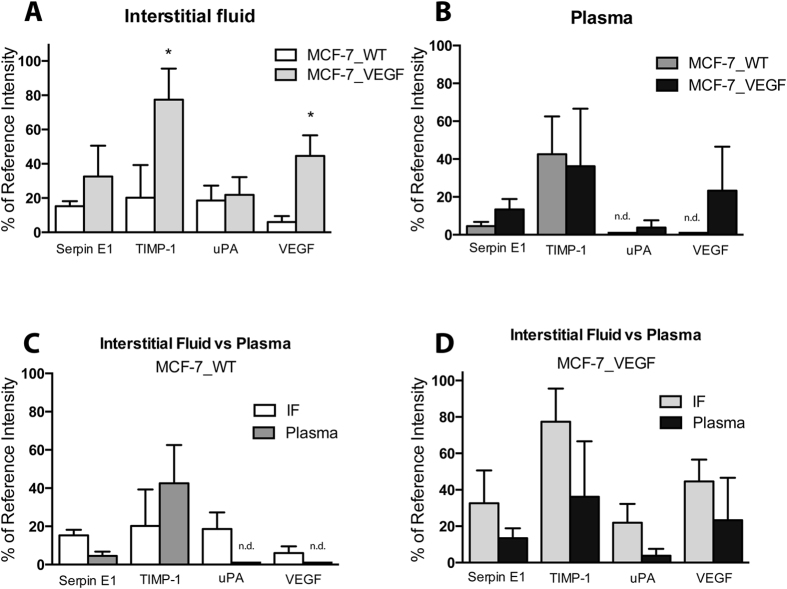
Angiogenesis-related factors in MCF-7 TIF and mouse plasma. (**A**) Angiogenic cytokines detected in the TIF of MCF-7_WT (n = 7) and MCF-7_VEGF (n = 13) tumors. (**B**) Angiogenic cytokines detected in the plasma of MCF-7_WT and MCF-7_VEGF tumors. (**C**) Comparison of angiogenic cytokines in TIF and plasma in the MCF-7_WT group. (**D**) Comparison of angiogenic cytokines in TIF and plasma in the MCF-7_VEGF group. Values represent Mean + SEM. *P < 0.05. nd = not detected.

To evaluate the angiogenic secretome, a Proteome Profiler Antibody Array Kit for Human Angiogenesis (R&D systems, Minnesota, USA) was used to determine 55 angiogenesis-related factors in TIF and plasma samples (Figure S1). As shown in Fig. 1A and B, from a panel of 55, only 4 factors (serpin E1, TIMP metallopeptidase inhibitor 1 (TIMP-1), urokinase plasminogen activator (uPA), and VEGF) were detectable in TIF derived from MCF-7_WT and MCF-7_VEGF tumors and the plasma from these tumor-bearing mice. VEGF overexpression in MCF-7 tumors resulted in a significant increase of VEGF and TIMP-1 and a trend towards an increase of serpin E1 and uPA in TIF (Fig. 1A). TIMP-1 was the only factor within the highest 50% range of the assay. Plasma samples from mice with MCF-7_WT tumors only contained detectable levels of serpin E1 and TIMP-1, but not uPA or VEGF. Plasma samples from mice with MCF-7_VEGF tumors contained detectable uPA and VEGF, and a trend towards higher serpin E1 (Fig. 1B). A comparison of cytokines between TIF and plasma in the MCF-7_WT tumor group (Fig. 1C) showed that although uPA and VEGF were detected in TIF, these were not detected in plasma, and there was a trend of lower serpin E1 and higher TIMP-1 in plasma compared to TIF. With VEGF overexpression, even though TIMP-1 increased significantly in TIF, this was not reflected as a detectable increase of plasma TIMP-1 levels, although increases of uPA and VEGF in TIF were also reflected in plasma (Fig. 1D).

### Angiogenesis-related factors in TIF and plasma of MDA-MB-231_WT and VEGF overexpressing tumors

**Figure 2:**
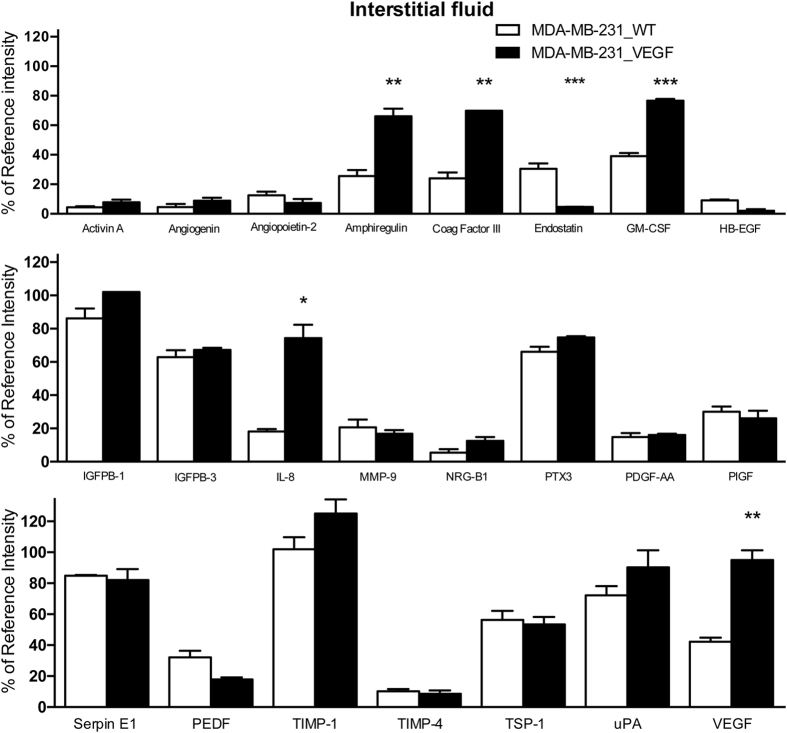
Angiogenesis-related factors in MDA-MB-231 TIF. Angiogenic cytokines detected in the TIF of MDA-MB-231_WT (n = 7) and MDA-MB-231_VEGF (n = 8) tumors. Values represent Mean + SEM. *P < 0.05; **P < 0.01; ***P < 0.001.

TIF from MDA-MB-231_WT tumors contained 23 detectable factors (activin A, angiogenin, amphiregulin, angiopoietin-2, coagulation factor III (Coag Factor III), endostatin, granulocyte monocyte colony-stimulating factor (GM-CSF), heparin-binding EGF-like growth factor (HB-EGF), insulin growth factor binding protein 1 (IGFBP-1), IGFBP-3, interleukin 8 (IL-8), matrix metallopeptidase 9 (MMP-9), neuregulin 1 (NRG1-β1), pentraxin 3 (PTX-3), platelet-derived growth factor AA (PDGF-AA), placental growth factor (PlGF), pigment epithelial-derived factor (PEDF or serpin F1), plasminogen activator inhibitor 1 (PAI-1 or serpin E1), TIMP-1, TIMP metallopeptidase 4 (TIMP-4), thrombospondin-1 (TSP-1), uPA, and VEGF) from the panel of 55 (Fig. 2) that also included the four factors detected in the TIF of MCF-7 tumors. Of the 23, IGFBP-1, IGFBP-3, PTX-3, serpin E1, TIMP-1, TSP-1 and uPA were within the highest 50% range of the assay.

VEGF overexpression resulted in a significant increase of 4 factors (amphiregulin, coagulation factor III, GM-CSF and IL-8) in addition to VEGF, and a decrease in endostatin (Fig. 2).

**Figure 3:**
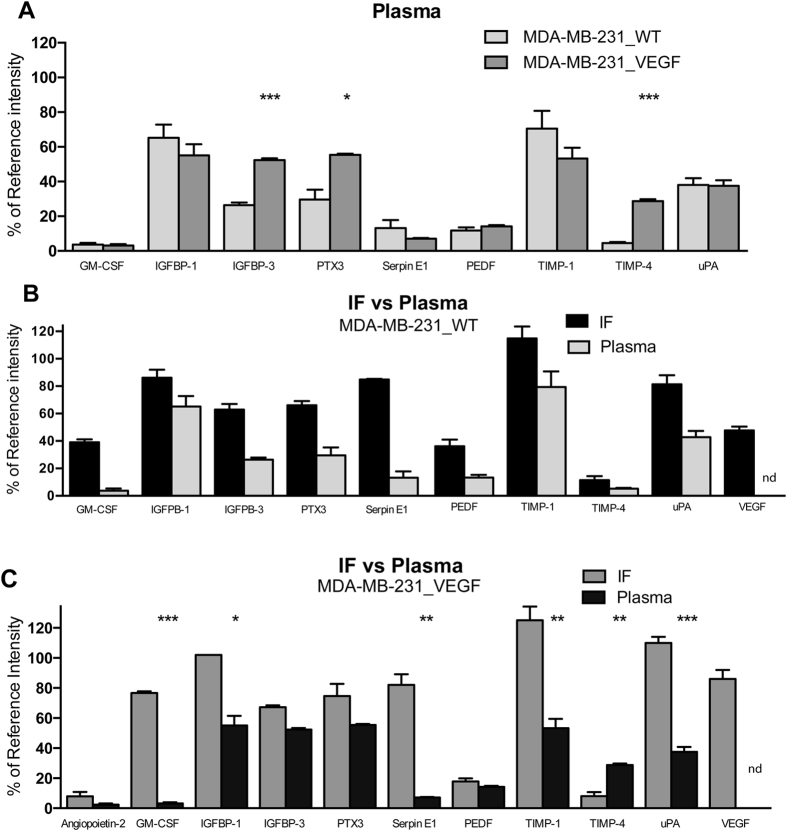
Angiogenesis-related factors in the plasma of MDA-MB-231 tumor-bearing mice and comparison to the TIF. (**A**) Angiogenic cytokines detected in the plasma of MDA-MB-231_WT (n = 7) and MDA-MB-231_VEGF (n = 8) tumors. Values represent Mean + SEM. *P < 0.05; ***P < 0.001. (**B**) Comparison of angiogenic cytokines in TIF and plasma in the MDA-MB-231_WT group. (**C**) Comparison of angiogenic cytokines in TIF and plasma in the MDA-MB-231_VEGF group. Values represent Mean + SEM. *P < 0.05; **P < 0.01; ***P < 0.001.

Several but not all of the factors observed in TIF were detected in the plasma (GM-CSF, IGFBP-1, IGFBP-3, PTX-3, PDGF-AA, serpin E1, PEDF, TIMP-1, TIMP-4 and uPA) of mice bearing MDA-MB-231_WT tumors (Fig. 3A). With VEGF tumor overexpression, IGFBP-3, PTX-3, and TIMP-4 increased significantly in the plasma (Fig. 3A).

Cytokines detected in plasma were found at lower levels than in TIF in the MDA-MB-231_WT group (Fig. 3B) and in the VEGF overexpressing group (Fig. 3C).

### Differential content in MCF-7 and MDA-MB-231 TIF and plasma

**Figure 4:**
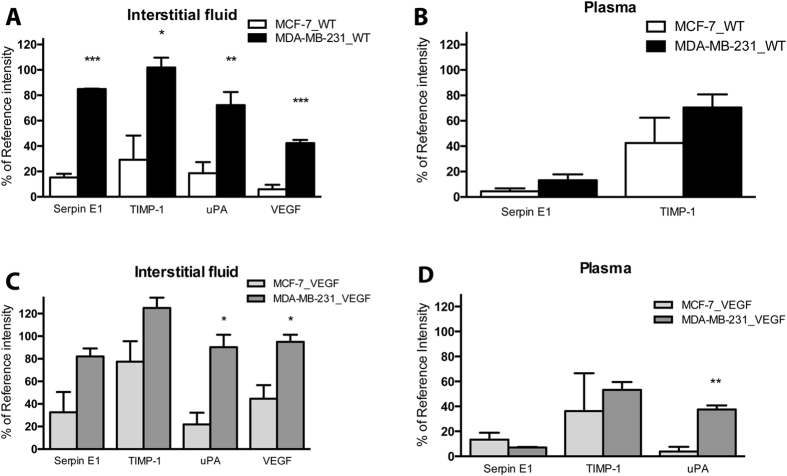
Comparison of MCF-7 and MDA-MB-231 TIF and plasma content. (**A**) Comparison of cytokines common to MCF-7_WT and MDA-MB-231_WT TIF. Of the 24 cytokines present in the MDA-MB-231 TIF, only serpin E1, TIMP-1, uPA and VEGF were found in MCF-7 TIF and at a significantly lower level. (**B**) Comparison of cytokines common to MCF-7_WT and MDA-MB-231 wt plasma. (**C**) Comparison of cytokines common to MCF-7_VEGF and MDA-MB-231_VEGF TIF. (**D**) Comparison of cytokines common to MCF-7_VEGF and MDA-MB-231_VEGF plasma. Values represent Mean + SEM. *P < 0.05; **P < 0.01; ***P < 0.001.

In addition to the 19 factors (activin A, angiogenin, amphiregulin, angiopoietin-2, coagulation factor III, endostatin, GM-CSF, HB-EGF, IGFBP-1, IGFBP-3, IL-8, MMP-9, NRG1-β1, PTX-3, PDGF-AA, PlGF, PEDF, TIMP-4 and TSP-1) detected in TIF from MDA-MB-231_WT tumors that were not detected in MCF-7_WT tumors, significant differences were also observed between the 4 proteins (serpin E1, TIMP-1, uPA and VEGF) that were detected in the TIF of both tumors. Those cytokines were significantly higher in MDA-MB-231_WT TIF compared to MCF-7_WT TIF (Fig. 4A). Compared to the 10 proteins (GM-CSF, IGFBP-1, IGFBP-3, PTX-3, PDGF-AA, serpin E1, PEDF, TIMP-1, TIMP-4 and uPA) detected in the plasma of MDA-MD-231_WT tumor bearing mice, only 2 (serpin E1 and TIMP-1) were detected in the plasma of MCF-7_WT tumor bearing mice. Both factors tended to be lower in MCF-7 plasma (Fig. 4B). A similar trend was observed when comparing the TIF cytokines for MCF-7_VEGF and MDA-MB-231_VEGF tumors. Differences for uPA and VEGF were significant (Fig. 4C). Plasma secretome comparison of VEGF overexpressing tumors showed no difference between MCF-7_VEGF and MDA-MB-231_VEGF tumors for TIMP-1, and a trend towards lower serpin E1 and significantly higher uPA in plasma from MDA-MB-231_VEGF tumor-bearing mice (Fig. 4D).

**Figure 5:**
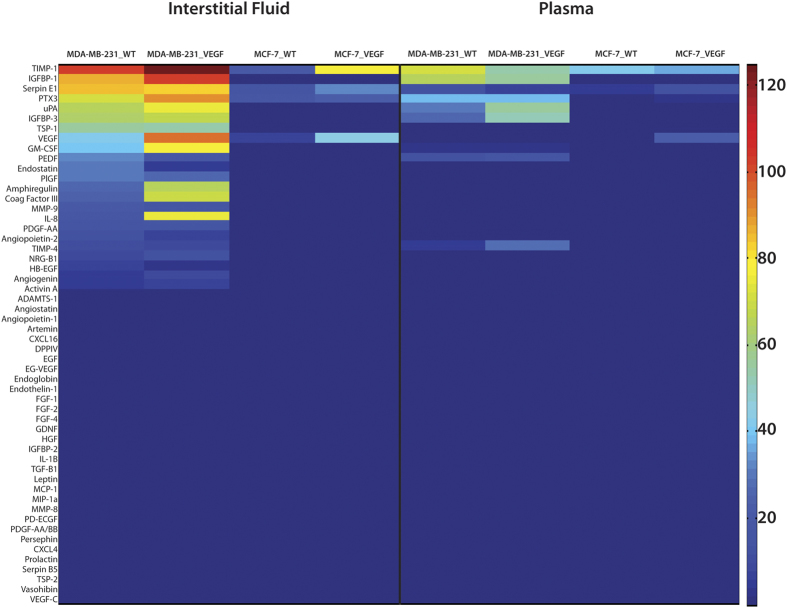
Heatmap of angiogenesis-related factors expression in TIF and plasma samples. Overview of the relative expression of angiogenesis-related cytokines in TIF (left, n = 7–8) and plasma (right, n = 7–13) of tumor-bearing mice for each cell type.

Angiogenesis factors detected in TIF and plasma of wild type and VEGF overexpressing tumors are summarized in the heat map in Fig. 5. The heat map visually confirms the secretome rich profile of MDA-MB-231_WT TIF compared to MCF-7_WT TIF, and the correspondingly lower number of factors in plasma from MCF-7_WT tumor bearing mice compared to MDA-MB-231_WT tumor bearing mice. VEGF overexpression resulted in an increase of a subset of factors in the TIF of MDA-MB-231_VEGF and MCF-7_VEGF tumors. Notably, amphiregulin, Coag Factor III, GM-CSF, IL-8, and VEGF increased significantly, whereas endostatin decreased significantly in MDA-MD-231 TIF following VEGF overexpression. TIMP-1 and VEGF were the two TIF factors that increased significantly with VEGF overexpression in MCF-7 tumors. The pattern of secretome changes observed in TIF following VEGF overexpression was not mirrored in the plasma secretome indicative of an active tumor utilization or regulation of TIF cytokines rather than passive diffusion into the plasma.

Analysis of conditioned media (CM) from these 4 cell lines identified patterns that were very different from the TIF data (Figure S2). Unlike TIF, CM from MCF-7_WT cells contained several factors. Of these, only serpin E1, TIMP-1 and VEGF were also detected in TIF, and uPA that was detected in TIF was absent in CM. Factors detected in CM from MCF-7_VEGF cells were similar to MCF-7_WT cells except for endostatin, which is consistent with TIF results, and suggests that cellular rather than tumor microenvironment processes caused the decrease of endostatin following VEGF overexpression. Also unlike TIF, the CM secretome was comparable between MCF-7_WT and MDA-MB-231_WT cells. Like CM medium from MCF-7 cells, CM from MDA-MB-231 cells was also comparable with or without VEGF overexpression, highlighting the role of the *in vivo* tumor microenvironment in the secretome.

### Quantitative analysis of VEGF content in MDA-MB-231 tumors

The overexpression of VEGF in the cells used here has been previously validated^27^. Quantitative ELISA of TIF samples (Figure S3) demonstrated a significant increase of VEGF in TIF from MDA-MB-231_VEGF tumors compared to MDA-MB-231_WT tumors (P < 0.05, n = 3).

## Discussion

Characterization of the angiogenic secretome in TIF and plasma of wild type and VEGF overexpressing ER-positive MCF-7 and triple negative MDA-MB-231 tumors provided new insights into differences in the angiogenic TIF and plasma secretome of these tumors, the relationship between the TIF and plasma secretomes, and the role of VEGF in modifying both. The TIF secretome was, for the most part, distinctly different from the plasma secretome, although some factors such as TIMP-1, IGFBP-1, PTX3, uPA and IGFBP-3 were observed in both TIF and plasma and may provide plasma biomarkers of the tumor secretome. Other factors that may play a role in the different patterns are the large blood volume that can dilute tumor-secreted factors, and the utilization of these factors by the tumor as well as other tissues. Because the chamber was excised as an endpoint we were unable to comment on pharmacokinetic behavior of the tumor produced proteins and can only discuss data from this single time-point measurement.

Profound differences in the TIF secretome between triple negative MDA-MB-231_WT and ER-positive MCF-7_WT tumors were observed. TIF from ER-positive MCF-7_WT tumors only contained low levels of serpin E1, TIMP-1, uPA and VEGF, and the plasma contained low levels of serpin E1 and TIMP-1. Triple negative breast cancer is significantly more aggressive and resistant to treatment^25^. The cytokine-rich TIF of MDA-MB-231 tumors compared to MCF-7 tumors may play a role in the aggressive phenotypic characteristics of these tumors. Twenty-three factors were detected in the TIF from MDA-MB-231 tumors, of which only 4 were detected in TIF from MCF-7 tumors. In addition to established pro-angiogenic factors, including angiopoietin-2, IL-8 and GM-CSF, MDA-MB-231 tumors also secreted angiogenesis inhibitors such as endostatin, serpin E1, PEDF and TSP-1. It has been suggested that even in highly angiogenic tumors, there is a fine balance between pro- and anti-angiogenic factors^28^.

In the triple negative MDA-MB-231_WT tumors, TIMP-1 followed by IGFBP-1 and serpin E1 were the cytokines at the highest concentration in TIF. In a previous study, characterization of CM from MDA-MB-231 cells using the identical assay, identified the presence of PEDF, VEGF, angiogenin, endothelin, HB-EGF, MMP9, PIGF, serpin E1, TIMP-1 and TSP-1^29^. In our study, we detected very different secretome profiles between CM obtained from cells in culture and TIF collected from tumors *in vivo*, highlighting the importance of the *in vivo* tumor microenvironment in the tumor secretome. Indeed, endothelin was not detected in TIF, while 15 other factors that were present in TIF were absent in the CM. These differences may be partly explained by an increased concentration of the cytokines in the TIF due to higher cell density and the accumulation of TIF in the small chamber volume *in vivo*. Crosstalk between cancer cells and stromal cells in the microenvironment such as endothelial cells may also induce the secretion of more factors by cancer cells^30^.

VEGF overexpression in stably transfected MCF-7 cells was associated with increased capillary growth and accelerated tumor growth^31^, and was correlated to altered vasculature in animal models^27^ and a poor clinical outcome^32^. Elevated tumor VEGF was therefore considered as a potential biomarker for therapy response^33^ and a possible effector of resistance to hormone treatment in ER-positive tumors^34^.

With VEGF overexpression, TIMP-1 and VEGF increased significantly in the TIF of MCF-7_VEGF tumors, and there was a trend towards an increase of serpin E1. However, the significant increase of TIMP-1 in TIF did not appear in the plasma of these mice, suggesting utilization, for instance through internalization or enzymatic processing, of this cytokine by cancer or stromal cells. Also, lymphatic clearance and a shorter half-life in plasma could play a role in the decrease or absence of those cytokines at detectable concentrations. Both VEGF and serpin E1 increased in the plasma of mice with VEGF overexpressing MCF-7 tumors, suggesting limited utilization of VEGF and serpin E1 by the cells.

Tissue inhibitor of metallopeptidase 1 (TIMP-1) has been identified as an inhibitor of angiogenesis^35^. TIMP-1 expression has been documented in luminal A breast cancer subtypes that include MCF-7 tumors^36^. TIMP-1 overexpression has also been observed to decrease expression of progesterone receptor, one of the major receptors in breast cancer cells, and mediate drug resistance in MCF-7 cells^37^,^38^ and promote tumor growth and cell survival^39^. Consistent with our observations that TIMP-1 significantly increased in TIF with VEGF overexpression in MCF-7 tumors, exogenous VEGF administration has been associated with TIMP-1 release in the retina^40^, although the regulation of TIMP-1 by VEGF is not fully understood. TIMP-1 is known to play a role in inflammation and immunity^41^, and its increase with VEGF overexpression is consistent with observations that VEGF potentiates immune suppression^42^.

Unlike ER-positive MCF-7 tumors where only TIMP-1 and VEGF were the two TIF cytokines that increased significantly with VEGF overexpression, amphiregulin, GM-CSF, IL-8, Coag Factor III, and VEGF increased significantly, whereas endostatin decreased significantly in TIF from triple negative MDA-MD-231 tumors following VEGF overexpression.

Concomitant regulation of amphiregulin, an EGF analog, and VEGF has been previously documented^43^. Synthesis of amphiregulin is triggered by hypoxia in breast carcinoma and other cancers in humans^44^. In that context HIF-2 induces both VEGF and amphiregulin in a concerted effort to promote angiogenesis^45^, which suggests that VEGF induced amphiregulin secretion in these cells that may in turn support its further synthesis in an autocrine/paracrine manner, similar to its action through EGFR. Indeed, self-sufficiency in the amphiregulin signaling cascade leading to its own synthesis has been widely demonstrated^43^. A better understanding of the dependence between amphiregulin and VEGF may lead to new treatments in triple negative breast cancers expressing VEGF. Amphiregulin has also been associated with the expression of other proteins observed in this study, such as MMP-9, uPA and serpin E1^46^,^47^.

GM-CSF is a major immunomodulator that is associated with the proliferation of granulocytes and monocytes. This cytokine has attracted attention for its potential to slow down tumor growth by an anti-angiogenic effect mediated by the secretion of soluble VEGF receptor 1 by macrophages^48^. It has demonstrated pro-angiogenic properties in rats *via* its action on VEGF and Tie-2^49^. GM-CSF has been detected in MDA-MB-231 conditioned media^50^ but is apparently absent from triple negative tumor samples in humans^51^. On the other hand, GM-CSF is considered an important mediator of angiogenesis and invasion in a wide variety of solid tumors^52^. However, the action of GM-CSF in breast cancer is not well defined and, outside of a role in bone metastases dissemination, very little is known on the role of GM-CSF. It will be important to identify the relationship between VEGF and GM-CSF and its role in mediating a more aggressive phenotype in VEGF expressing triple negative breasts cancers.

IL-8 and coagulation factor III also significantly increased in MDA-MB-231_VEGF tumors. IL-8 is a known pro-angiogenesis factor as early experiments using IL-8 transfection showed increased angiogenesis *in vivo* for gastric carcinoma^53^. Blockade of the IL-8 receptor CXCR2 decreased tumor proliferation and angiogenesis in metastatic melanoma^54^. IL-8 is thus increasingly being considered as a potential biomarker for aggressive breast cancer^51^. Our data have identified, for the first time, the dependence of IL-8 and coagulation factor III on VEGF that merit further investigation.

Another observation, made here for the first time, was the significant decrease of endostatin, a derivative of collagen XVIII, in TIF with VEGF overexpression in MDA-MB-231 tumors. As an anti-angiogenic agent, endostatin has been investigated as a therapeutic agent as part of combined therapy in breast cancer^55^,^56^. Recombinant human endostatin is under phase II and III clinical investigation for several types of cancer (clinicaltrials.gov). This unexpected finding requires further investigation and highlights the complex relationship between pro- and anti-angiogenic factors and the importance of understanding these interactions to achieve effective treatments.

Significant differences observed between the patterns of cytokines in the plasma and TIF confirmed the integrity of the chamber and the ability of the methodology to sample TIF. It is also important to consider some of the limitations of the model system used. The Proteome Profiler Array was designed to detect human proteins and therefore the factors detected are from the cancer cells and not mouse stromal cells present within the tumor. Since we used immunodeficient mice to generate human breast cancer xenografts, the contribution of functional T and B lymphocytes to the angiogenic TIF and plasma secretome was not investigated. Future studies with syngeneic tumor models or humanized mice may be able to evaluate the contribution of the immune system. Additionally, the tumors were implanted subcutaneously and not orthotopically, because of the limitations imposed by the chamber implantation. It is possible that orthotopic implantation may modify the TIF and plasma secretome and future studies should focus on using orthotopic models. The chamber we used collected TIF over the entire duration of tumor growth. It is possible that draining the chamber at time intervals would allow insights into dynamic changes in the TIF with tumor growth. The current constraints of using xenografts in mice limited the retrievable volume but as more sensitive assays evolve, such studies may become achievable.

These studies are forerunners of future work focusing on characterizing the entire TIF and plasma secretome in triple negative breast cancers, within the context of the ER/PR status of breast cancers, to understand the mechanisms that drive the aggressiveness of breast cancers and their resistance to treatment. Such studies can be achieved by intraoperatively collecting TIF for analysis. Such samples can also be used to identify the aggressiveness of the cancer that may assist in staging and applying precision medicine in breast and other lethal cancers. These rich data samples can be mined for identifying new treatment strategies and can be incorporated into Systems Biology based computational approaches to design effective personalized treatments.

## Materials and Methods

### Construction of the TIF collection chamber

To build the TIF collection chamber (Figure S4A), modified from previous work by Gullino^2^, a thin layer of biocompatible USP Class VI epoxy-type sealant EP21LVMED (Masterbond, New Jersey, USA) was coated on one rim of a nylon 6,6 spacer, outer diameter 6.4 mm, length 3.2 mm, screw size 8, inner volume 45 μl (part #94639A101, McMaster-Carr, New Jersey, USA). A 0.45 μm MF-Millipore Membrane filter with 13 mm diameter (cat. #HATF01300, EMD Millipore, Massachusetts, USA) was carefully placed on the sealant and the sealant was allowed to cure and dry for 12 hours. The same procedure was repeated for the other rim of the spacer. Each chamber was visually inspected to assure the tightness of the seal. Where necessary, additional sealant was applied and the curing process was repeated. The chambers were sterilized with ethylene oxide at 65 °C and preserved under dehumidified conditions until use.

### Cancer cell lines and culture

MCF-7 and MDA-MB-231 cells overexpressing VEGF were generated and characterized as previously described^27^. Expression of VEGF in cells was quantified prior to tumor implantation using a Human VEGF Quantikine Elisa Kit (DVE00, R & D systems, Minnesota, USA).

### MDA-MB-231 and MCF-7 xenografts

Female severe combined immunodeficient (SCID) mice, aged 4–6 weeks, were used in these studies. The numbers of mice used per group of tumors from which TIF and plasma were sampled were: MCF-7 wild type (MCF-7_WT, n = 7), MCF-7 with VEGF overexpression (MCF-7_VEGF, n = 13), MDA-MB-231 wild type (MDA-MB-231_WT, n = 7) and MDA-MB-231 with VEGF overexpression (MDA-MB-231_VEGF, n = 7). All animals were handled in accordance with good animal practice, and all animal work was conducted under a protocol approved by the Institutional Animal Care and Use Committee (IACUC) of Johns Hopkins University. Therefore, all procedures were performed according to the relevant guidelines and regulations dictated by the IACUC. The Johns Hopkins University animal facility is accredited by the American Association for the Accreditation of Laboratory Animal Care and meets National Institute of Health standards as set forth in the “Guide for the Care and Use of Laboratory Animals” (DHHS Publication No. (NIH) 85–23, Revised 1985).

### Surgical Procedure and TIF collection

To produce tumor pieces for TIF collection chamber implantation, two mice were injected subcutaneously in the flank with 2 × 10^6^ cells for the cell lines used. Mice inoculated with MCF-7_WT and MCF-7_VEGF cells were implanted with a subcutaneous pellet of estradiol 24 hours prior to cell inoculation (0.18 mg of 17β-Estradiol, 60-day release, Innovative Research of America, Florida, USA). Mice were monitored weekly until the tumor volumes were 250–500 mm^3^. The tumor was excised, and placed in a Petri dish containing Hanks buffered saline solution (HBSS).

To obtain tumors that incorporated a TIF collection chamber, mice were anesthetized with a mixture of acepromazine (25 mg/kg) and ketamine (2.5 mg/kg), and the flank was shaved and swabbed with ethanol. A minimal incision was made and bluntly expanded into a pocket. A sterile collection chamber was placed within this pocket along with 4 small (approx. 1 mm^3^) tumor pieces evenly spread around the chamber. The site was closed with sterilized wound clips. The entire procedure was performed under sterile conditions. The tumor was allowed to grow until it encapsulated the chamber and the TIF drained into the chamber. Once the tumor with the encapsulated chamber reached ~500 mm^3^, the mouse was euthanized and the tumor excised without disturbing the chamber. Using a scalpel, a small incision was made in the tumor to reach the filter surface of the chamber. The membrane was punctured with a 10 μl pipette tip and the IF (approximately 45 μl per chamber) was removed and immediately frozen at −80 °C for further analyses. Representative examples of a prepared chamber, a chamber within the tumor, and the TIF sampled are shown in Figures S4A–C, respectively.

Plasma samples were collected from the inferior vena cava of terminally anesthetized mice just prior to euthanasia and TIF retrieval.

### Cytokine analysis

Proteome Profiler Antibody Array kits for human angiogenesis factors (R&D Systems, Minnesota, USA) were used according to the manufacturer’s instructions to identify cytokines in the TIF and plasma. For each experiment, 50 μl of pooled TIF or plasma was diluted in 1.5 ml of the array buffer. For CM, 1.0 ml was mixed with 0.5 ml of array buffer. The TIF from MCF-7_WT, MCF-7_VEGF, MDA-MB-231_WT and MDA-MB-231_VEGF tumors were compared for IF and corresponding plasma samples. Each membrane was analyzed using ImageJ 1.49 u. Supplementary Figure 1 (Figure S1) shows typical membrane staining for TIF from each type of tumor. The relative intensity of each dot, reflecting the concentration, was expressed in percentage of the reference values (average of all three reference spots on each membrane, see Figure S1) measured for each membrane. The experiments were performed in triplicates for statistical analyses. Cytokine levels under 2.0 in relative intensity were considered not detectable (nd).

### Statistical analysis

Unless otherwise specified, all statistical analyses were performed using a two-sided unpaired t-test using Graphpad Prism 6 (Graphpad, La Jolla CA). A *p*-value of 0.05 or less was considered significant. The n number represents the number of animals from which the pooled samples were obtained. Pooled samples were analyzed in three separate experiments for which the statistical analyses were performed using a sample size of 3.

## Additional Information

**How to cite this article:** Dore-Savard, L. *et al*. The Angiogenic Secretome in VEGF overexpressing Breast Cancer Xenografts. *Sci. Rep.* **6**, 39460; doi: 10.1038/srep39460 (2016).

**Publisher's note:** Springer Nature remains neutral with regard to jurisdictional claims in published maps and institutional affiliations.

## Supplementary Material

Supplementary Figures
