# Supplementary material for: The Angiogenic Secretome in VEGF overexpressing Breast Cancer Xenografts
Source: Sci Rep. 2016 Dec 20;6:39460. doi: 10.1038/srep39460 (PMC5171865; doi:10.1038/srep39460)
Supplement: Supplementary Figures [file srep39460-s1.pdf]

The Angiogenic Secretome in VEGF overexpressing Breast Cancer Xenografts

Louis Dore-Savard[1], Esak Lee[2], Samata Kakkad[1], Aleksander S. Popel[2,3] and Zaver M. Bhujwalla[1,3*]

[1]JHU ICMIC Program, Division of Cancer Imaging Research, The Russell H. Morgan Department of Radiology and Radiological Science;
[2]Systems Biology Laboratory, Department of Biomedical Engineering; [3]Sidney Kimmel Comprehensive Cancer Center
The Johns Hopkins University School of Medicine, Baltimore MD, USA

- **Supplementary information -**

Figures S1-S4 and legends

Figure S1. Representative images of the array. Reverse western assays with the human angiogenesis antibody arrays (R&D systems) were used to detect relative amounts of 55 factors in TIF from MCF-7_WT, MCF-7_VEGF, MDA-MB-231_WT and MDA-MB-231_VEGF tumors and in plasma from these mice. Factors that signficantly changed following VEGF overexpression are boxed and labeled. Ref = Reference spots, AR = Amphiregulin, CFIII = coagulation factor III.

Figure S2. Angiogenesis-related factors in conditioned media of breast cancer cell lines. Angiogenic factors detected in conditioned media from MCF-7_WT, MCF-7_VEGF, MDA-MB-231_WT and MDA-MB-231_VEGF cell lines.

Figure S3. **Quantitative analysis of VEGF content.** VEGF protein concentration (pg/ml) in TIF from MDA-MB-231 wt and MDA-MB-231_VEGF tumors (n=3). Values represent Mean $\pm$ SEM. * $P < 0.05$.

Figure S4. Tumor interstitial fluid collection in mice. A) Collection chamber made of nylon 6,6 tubing and a filter membrane on each end. The outer diameter is 6.35 mm and inner volume is 45 µl. B) Representative MDA-MB-231 tumor containing the collection chamber 5 weeks after implantation. The tumor completely encapsulated the chamber. C) Representative example of TIF collected from the chamber.

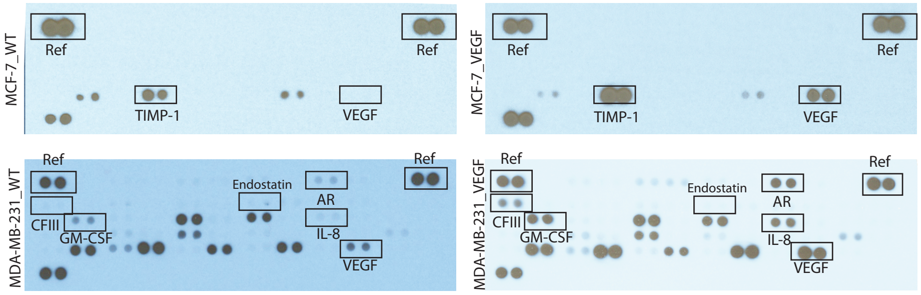
MCF-7_WT
Ref
Ref
TIMP-1
VEGF
MCF-7_VEGF
Ref
Ref
TIMP-1
VEGF
MDA-MB-231_WT
Ref
Ref
Endostatin
AR
CFIII
GM-CSF
IL-8
VEGF
MDA-MB-231_VEGF
Ref
Ref
Endostatin
AR
CFIII
GM-CSF
IL-8
VEGF

Figure S1

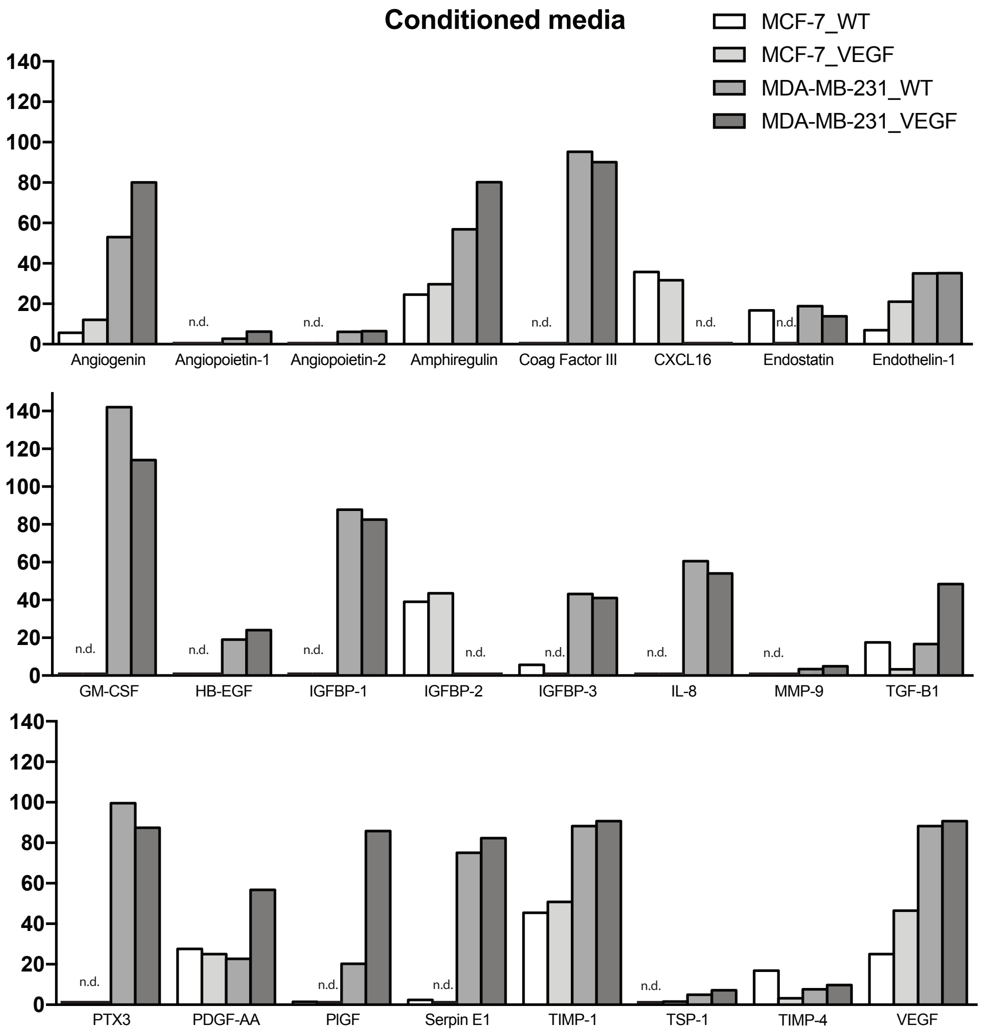
Conditioned media
MCF-7_WT
MCF-7_VEGF
MDA-MB-231_WT
MDA-MB-231_VEGF
140
120
100
80
60
40
20
0
n.d.
Angiogenin
Angiopoietin-1
Angiopoietin-2
Amphiregulin
Coag Factor III
CXCL16
Endostatin
Endothelin-1
GM-CSF
HB-EGF
IGFBP-1
IGFBP-2
IGFBP-3
IL-8
MMP-9
TGF-B1
PTX3
PDGF-AA
PlGF
Serpin E1
TIMP-1
TSP-1
TIMP-4
VEGF

Figure S2

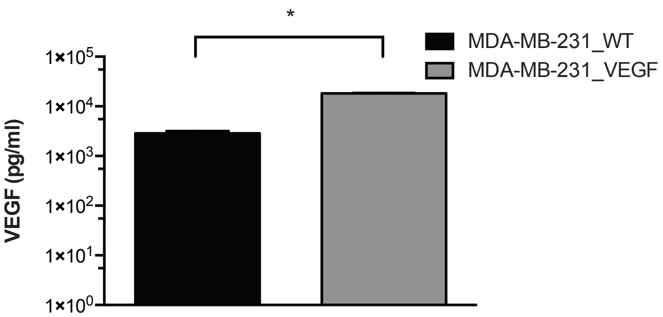
*
MDA-MB-231_WT
MDA-MB-231_VEGF
1×10^5
1×10^4
1×10^3
1×10^2
1×10^1
1×10^0
VEGF (pg/ml)

Figure S3

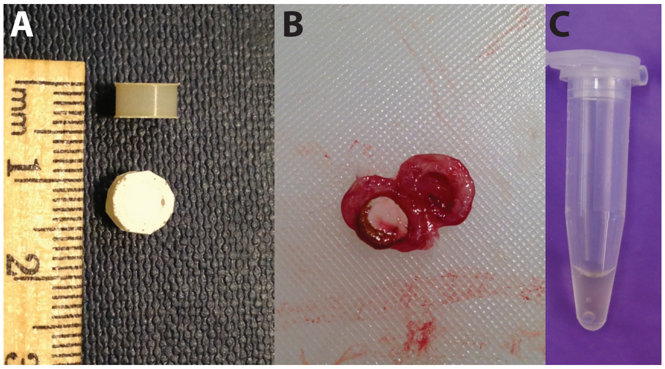

Figure S4
